# Solving Strength–Toughness Dilemma in Superhard Transition-Metal Diborides via a Distinct Chemically Tuned Solid Solution Approach

**DOI:** 10.34133/research.0035

**Published:** 2023-01-16

**Authors:** Xinlei Gu, Chang Liu, Xinxin Gao, Kan Zhang, Weitao Zheng, Changfeng Chen

**Affiliations:** ^1^ State Key Laboratory of Superhard Materials, Department of Materials Science and Key Laboratory of Automobile Materials, MOE, Jilin University, Changchun 130012, China.; ^2^ International Center for Computational Methods & Software, College of Physics, Jilin University, Changchun 130012, China.; ^3^ Department of Physics and Astronomy, University of Nevada, Las Vegas, NV 89154, USA.

## Abstract

Solid solution strengthening enhances hardness of metals by introducing solute atoms to create local distortions in base crystal lattice, which impedes dislocation motion and plastic deformation, leading to increased strength but reduced ductility and toughness. In sharp contrast, superhard materials comprising covalent bonds exhibit high strength but low toughness via a distinct mechanism dictated by brittle bond deformation, showcasing another prominent scenario of classic strength–toughness tradeoff dilemma. Solving this less explored and understood problem presents a formidable challenge that requires a viable strategy of tuning main load-bearing bonds in these strong but brittle materials to achieve concurrent enhancement of the peak stress and related strain range. Here, we demonstrate a chemically tuned solid solution approach that simultaneously enhances hardness and toughness of superhard transition-metal diboride Ta_1−*x*_Zr*_x_*B_2_. This striking phenomenon is achieved by introducing solute atom Zr that has lower electronegativity than solvent atom Ta to reduce the charge depletion on the main load-bearing B–B bonds during indentation, leading to prolonged deformation that gives rise to notably higher strain range and the corresponding peak stress. This finding highlights the crucial role of properly matched contrasting relative electronegativity of solute and solvent atoms in creating concurrent strengthening and toughening and opens a promising avenue for rational design of enhanced mechanical properties in a large class of transition-metal borides. This strategy of concurrent strength–toughness optimization via solute-atom-induced chemical tuning of the main load-bearing bonding charge is expected to work in broader classes of materials, such as nitrides and carbides.

## Introduction

Materials with superior mechanical strengths are crucial to many areas of modern industries and the scientific enterprise by providing cutting and drilling tools, structural components, protective coatings, and abrasives that find wide-ranging applications. Latest advances in manufacturing and operation of diverse equipment and devices raise pressing demands for materials that can withstand more complex and extreme loads. Of particular interest is a class of superhard materials with indentation hardness above 40 GPa that meet the increasingly stringent performance requirements [1–4]. A major impediment, however, is that rising hardness is usually accompanied by embrittlement, which causes material deterioration and even abrupt structural failure at high working loads. An urgent task is to enhance toughness, defined as ability to resist crack initiation and propagation while maintaining superior strength, but it faces the major challenge of the long-established hardness–toughness tradeoff dilemma in materials research [5]. Recent years have seen fast increasing research and development of transition-metal diborides (TMB_2_) that exhibit superhard nature stemming from the high electron density from the TM atoms, which help raise the bulk modulus in resistance to compression, and covalent bonding network formed by boron atoms, which contributes to enhanced ability to sustain shear strain leading to high hardness [6–8]. Moreover, TMB_2_ are inexpensive to synthesize compared to traditional superhard materials such as diamond and cubic boron nitride [9,10]. The TMB_2_ compounds also possess excellent properties such as high melting point, thermal stability, chemical inertness, thermal conductivity, and corrosion resistance, making these compounds suitable for most demanding applications in high-temperature environments [11].

A series of work in recent years has established a number of superhard compounds in the TMB_2_ family. A prominent early case is ReB_2_, which was synthesized in bulk quantities via arc melting at ambient pressure [12]. Microindentation measurements showed that the ReB_2_ possessed an average hardness of 48.0 GPa under an applied load of 0.49 N and made scratches on the surface of diamond [12]. A later work revealed that HfB_2_ and TaB_2_ films also reached superhard category with indentation hardness values of about 44.0 and 43.9 GPa, respectively, on specimens in their preferred growth (001) orientation [13]. Most recently, we explored the orientation-dependent superhardness of TaB_2_ film and found that load-constrained deformation improves dynamic stability and leads to enhanced strength of TaB_2_ (001) film, which was confirmed by an experimentally measured indentation hardness of 45.9 GPa [14].

Extensive research has aimed to further enhance hardness of TMB_2_ compounds by constructing dual-TMB_2_ solid solutions, as reported for Ti_0.57_W_0.43_B_2−*z*_ that has a higher hardness of 38.5 GPa compared to TiB_2_ (30.7 GPa) [15], V_0.69_W_0.21_B_2_ that has a higher hardness of 39.5 GPa compared to VB_2_ (33.9 GPa) [16], and Zr_0.8_Ta_0.2_B_1.8_ that has a higher hardness of 42.3 GPa compared to ZrB_2_ (35.0 GPa) [17]. While solid solution strengthening is well established and widely used in metals, where solute atoms are introduced to create local distortions in base crystal lattice and impede the plastic deformation caused by dislocation motion, the hardening phenomena in dual-TMB_2_ solid solutions are attributed to various distinct structural factors, including film growth direction and reduced crystal column size [15], dense structure [16], and age hardening during high-temperature annealing [17]. Meanwhile, there has been a general lack of study and understanding on the toughness of dual-TMB_2_ solid solutions, and it remains unclear whether enhanced toughness can be concurrently achieved along with increased hardness. This is an interesting and challenging problem because the strength–toughness tradeoff dilemma is known to impact a broad variety of material classes, from metals to ceramics, but different classes of materials are affected by distinct mechanisms. In metals, for instance, it is known that plastic deformation is dominated by dislocation motion, and solid solution strengthening impedes dislocation motion, rendering lower ductility and reduced toughness. Superhard materials, on the other hand, comprise strong, pure or mixed, covalent bonds that can sustain large peak stress, giving rise to high strength, but usually only support small strains, leading to brittle bond rupture past the peak stress and initiating plastic deformation dominated by crack formation and propagation. Such brittle nature stems from local bond deformation and breaking modes, so a remedy to the strength–toughness tradeoff in dual-TM borides needs to find a way to tune the chemical character of the main load-bearing B–B bonds. Valence electron concentration is known to impact mechanical properties of TM compounds [18,19]; recent study further identified relative electronegativity (REN) of constituent atoms in dual-TMB_2_ as a key factor in describing the variation trends in elastic moduli [20]. It remains, however, unclear how REN will affect stress response at large strains, where ultimate material strength and toughness are determined by the peak stress and related strain range under the measurement (indentation) deformation. In particular, little is known about intrinsic mechanisms of solid solution strengthening and toughening that would work for TMB_2_ compounds at the atomic bonding level.

While the conceptual design of REN was originally developed to describe material properties based on elastic responses to small strains [20], the insights remain relevant to understanding structural and property changes at large strains, especially the relative bonding charge variations among the constituent atoms under large deformations. On the basis of this consideration, we take Ta_1−*x*_Zr*_x_*B_2_ as an exemplary case study to explore the effect of tuning the bonding charge on main load-bearing B–B bonds by introducing solute Zr atoms to substitute for a portion of solvent Ta atoms. It is expected that the lower electronegativity (EN) of Zr (EN = 1.33) would weaken the ability to attract electrons compared to Ta (EN = 1.5) in the original TaB_2_ crystal, thereby allowing more bonding charge to be retained on the B–B bonds during the indentation-induced deformation. This scenario may extend the strain range of the deformed crystal to enhance toughness, while the extended strain range may generate higher peak stress to enhance strength. This idea represents a distinct chemically tuned solid solution approach that could produce concurrent strengthening and toughening in TM compounds. To validate this idea and elucidate the underlying mechanism, we carried out pertinent experimental and computational studies. We employed magnetron cosputtering technique to deposit TaB_2_, ZrB_2_, and Ta_1−*x*_Zr*_x_*B_2_ solid solution films in the preferred growth (001) orientation and then performed systematic indentation measurements to evaluate the hardness and toughness of these films. The results show that the synthesized Ta_3_ZrB_8_ solid solution film possesses superior hardness and toughness compared to TaB_2_ and ZrB_2_ films, demonstrating the desired concurrent strength–toughness enhancement. Computational simulations and related analysis reveal that, compared to Ta atoms in the TaB_2_ crystal, the substituted Zr atoms with lower EN cause a notable reduction in the amount of charge redistribution from the surrounding B–B bonds during indentation deformation, leading to enhanced B–B bonds that are able to sustain longer deformation strain range along with higher peak stress. The results of these joint experimental and computational studies confirm the expectations of the designed chemically tuned solid solution approach, showcasing the crucial role of solute atoms with properly contrasting EN in simultaneously strengthening and toughening dual-TMB_2_. The insights gained from the present study offer a useful guide for rational design and development of strong and tough materials among a large class of TM compounds.

## Results and Discussion

### Structural and physical properties: Experiments

**Fig. 1.**
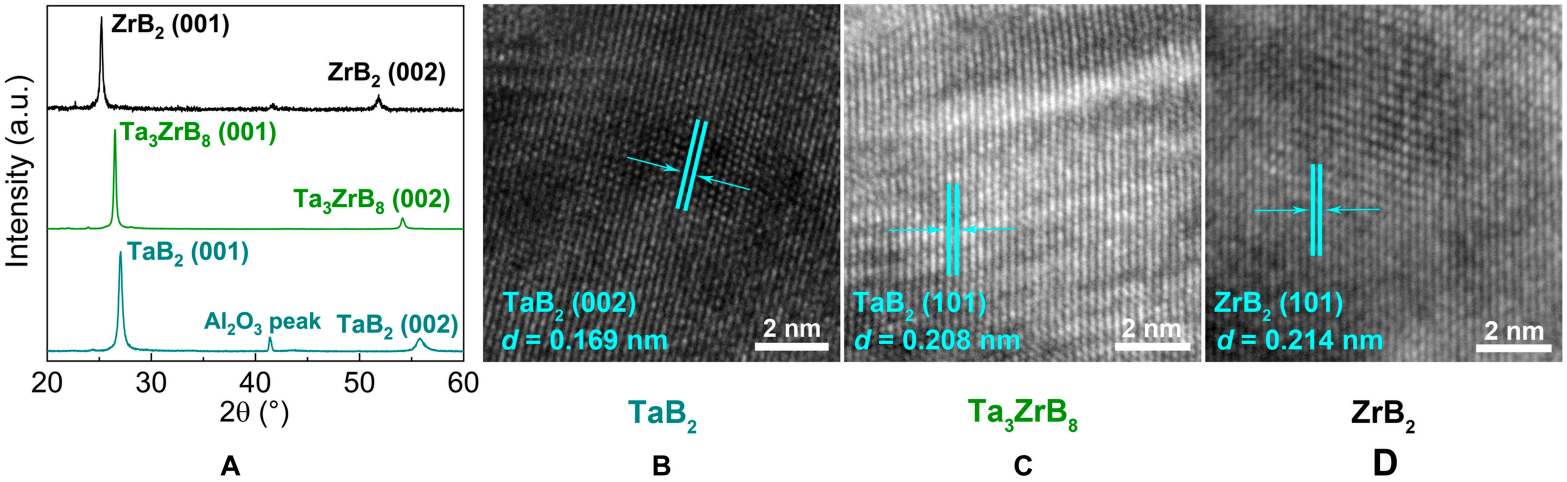
(A) The measured x-ray *θ*/2*θ* scan patterns. (B to D) HRTEM images of the as-deposited TaB_2_, Ta_3_ZrB_8_, and ZrB_2_ films, with selected lattice spacings indicated. a.u., arbitrary units.

We present the x-ray diffraction (XRD) *θ*-2*θ* scan patterns of TaB_2_, Ta_3_ZrB_8_, and ZrB_2_ films in Fig. 1A. Three diffraction peaks are observed in the XRD pattern of TaB_2_ film [power diffraction file (PDF): 65-0878], where the TaB_2_ (001), Al_2_O_3_ substrate [21], and TaB_2_ (002) peaks are seen at 2*θ* = 27.7°, 41.9°, and 56.5°, respectively. The sharp TaB_2_ (001) and (002) peaks indicate that the synthesized TaB_2_ film exhibits a strong preferred (001) growth orientation. Similar phenomena are also found in the XRD pattern of the ZrB_2_ film (PDF: 65-3389), which displays 3 diffraction peaks at 2*θ* = 25.2°, 41.9°, and 51.8°, corresponding to ZrB_2_ (001) peak, Al_2_O_3_ substrate peak, and ZrB_2_ (002) peak, respectively, indicating the strong (001) preferred orientation of the ZrB_2_ film. With Zr atoms (atomic radius, 145 pm) substituting for Ta atoms (atomic radius, 134 pm) in the TaB_2_ crystal, a lattice expansion occurs, resulting in a lower-angle shift of the diffraction peak in the Ta_3_ZrB_8_ film compared to that of the TaB_2_ film. Moreover, only 2 peaks are observed in the XRD pattern of the Ta_3_ZrB_8_ film: Ta_3_ZrB_8_ (001) and (002) peaks at 2*θ* = 26.5° and 54.1°, respectively, suggesting that the Ta_3_ZrB_8_ solid solution film has also strong (001) preferred orientation. The TaB_2_, Ta_3_ZrB_8_, and ZrB_2_ films all adopt the hexagonal crystal structure with the *P*6*/mmm* space group symmetry. The full widths at half maximum of the (001) peaks for the TaB_2_, Ta_3_ZrB_8_, and ZrB_2_ films are 0.44°, 0.24° and 0.29°, respectively, giving the estimated grain sizes of ~20, ~33, and ~28 nm using the Scherrer formula [22]. The high-resolution transmission electron microscopy (HRTEM) images of the TaB_2_, Ta_3_ZrB_8_, and ZrB_2_ films are shown in Fig. 1B and C, which verify their crystal structures. The interplanar spacing *d* values in Fig. 1B and D are measured to be 0.169 and 0.214 nm, which are close to the *d* values of TaB_2_ (002) lattice plane (*d*_002_ = 0.164 nm) and ZrB_2_ (101) lattice plane (*d*_101_ = 0.216 nm) given in PDF cards. Figure 1C exhibits a *d* value of 0.208 nm for the Ta_3_ZrB_8_ (101) plane, and this value is smaller than *d*_101_ = 0.216 nm of ZrB_2_ (101) plane and slightly larger than *d*_101_ = 0.206 nm of TaB_2_ (101) plane, indicating that these *d* values follow Vegard’s law, which is often obeyed by solid solution crystal structures.

**Fig. 2.**
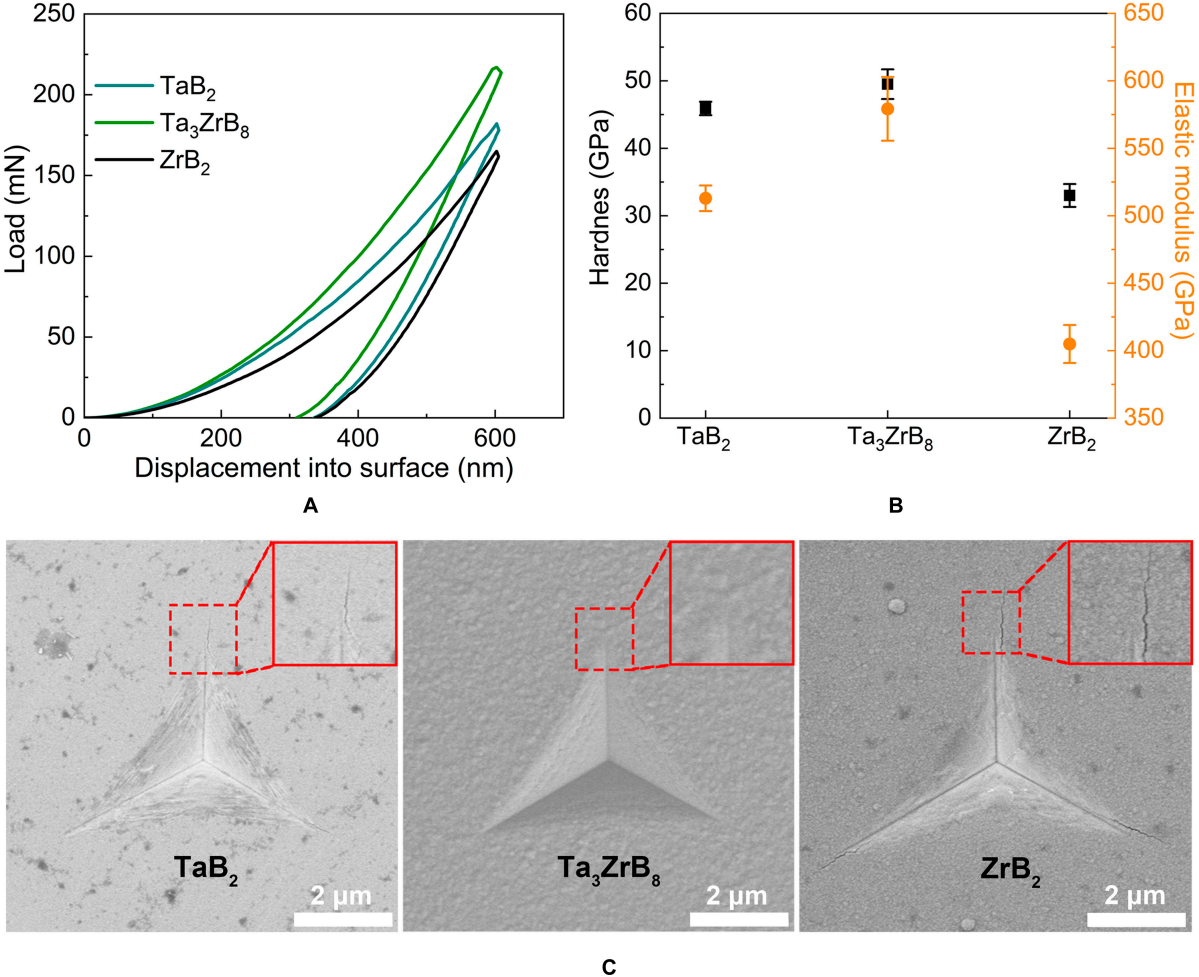
(A) The experimental loading–displacement curves, (B) the hardness and elastic modulus data, and (C) the indentation site morphology of the TaB_2_, Ta_3_ZrB_8_, and ZrB_2_ films with the amplified views of the presence/absence of cracks at the tip of the indentation imprint shown in the top-right corners of each panel.

The mechanical properties of TaB_2_, Ta_3_ZrB_8_, and ZrB_2_ films are characterized by means of nanoindentation device equipped with Berkovich diamond indenter. Figure 2A displays the measured load–displacement curves for these synthesized films. The forces loaded on the Berkovich diamond indenter at an indentation depth of 600 nm are 181.9, 217.4, and 164.9 mN for the TaB_2_, Ta_3_ZrB_8_, and ZrB_2_ films, respectively, in which the load force on the Ta_3_ZrB_8_ film is the highest, indicating that the deformation resistance of the Ta_3_ZrB_8_ film is stronger than those of the TaB_2_ and ZrB_2_ films. The hardness values of the TaB_2_, Ta_3_ZrB_8_, and ZrB_2_ films were estimated using the Oliver–Pharr method [23,24], and the results were evaluated from the indentation depth range of 50 to 120 nm (Fig. S1), producing the hardness values of 45.9 ± 1.0, 49.5 ± 2.2, and 33.0 ± 1.7 GPa for the TaB_2_, Ta_3_ZrB_8_, and ZrB_2_ films (Fig. 2B), respectively. These results show that the formation of Ta_3_ZrB_8_ solid solution enhances the hardness of superhard TaB_2_. The elastic modulus of each sample can be obtained from the slope at the initial position of the unloading section [23] of the load–displacement curves in Fig. 2A, and the values are 512.9 ± 9.5, 579.2 ± 23.6, and 404.9 ± 14.1 GPa for the TaB_2_, Ta_3_ZrB_8_, and ZrB_2_ films, respectively, as also shown in Fig. 2B. Scanning electron microscope (SEM) morphology images in Fig. 2C reveal that indented TaB_2_ and ZrB_2_ films show radial cracks, while no cracks are seen around the indentation site of the Ta_3_ZrB_8_ film, showing enhanced toughness of the Ta_3_ZrB_8_ film compared to the TaB_2_ and ZrB_2_ films and demonstrating concurrent strengthening and toughening of the Ta_3_ZrB_8_ solid solution. We have checked the data for all 9 indentation points of each film and got the same toughness variation tendency. It is noted that nanoindentation is known to produce higher hardness than microindentation. However, the nanoindentation method is still an appropriate way to characterize the true hardness of thin film materials, because we must eliminate the substrate effect when the film materials are only a few micrometers. Besides, thin-film materials prepared by physical vapor sputtering usually occur good crystalline quality and high densification. Hence, the thin film materials should exhibit a higher hardness than the bulk materials, which usually achieved a slightly lower densification due to the introduction of pores during the sintering. We would like to emphasize that the solid solution strengthening and toughening effects were found in the transverse comparison concerning the mechanical properties of the 3 films using the unified test method.

**Fig. 3.**
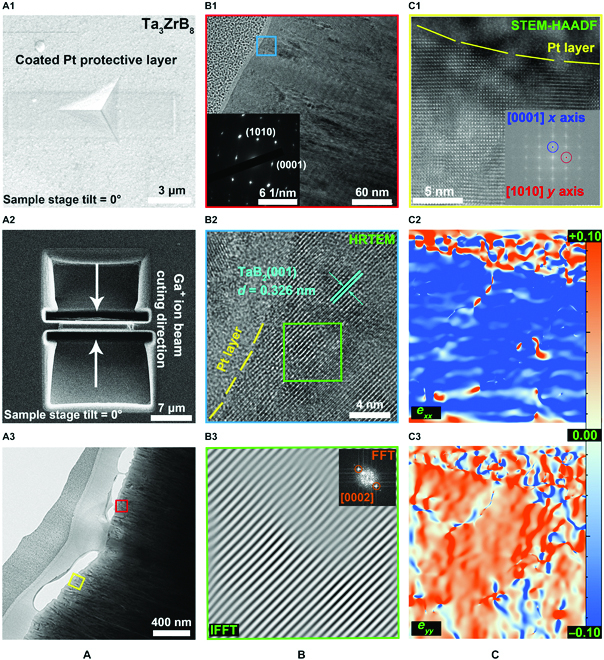
(A) The schematic diagram of FIB sample preparation process and cross-sectional TEM image of the indented Ta_3_ZrB_8_ film. (B) An enlarged view of the area marked by the red box in (A3), and the insert is an selected-area electron diffraction pattern in a circular area 126 nm in diameter beneath the indentation surface. The HRTEM image around the indentation surface is marked by the blue square in (B1), and the boundary between the indentation surface and a Pt layer is marked by the yellow dashed line. The fast Fourier transform and inverse fast Fourier transform images of the zone labeled by the green box in HRTEM image to analyse the [002]-type dislocation distribution beneath the indented surface. (C) The STEM-HAADF picture beneath the indentation surface concerning the yellow box in (A3), the corresponding geometric phase analysis results of STEM-HAADF picture declare a complex strain mode of Ta_3_ZrB_8_ under berkovich indentation deformation.

To further assess structural changes under indentation, we prepared a cross-sectional sample of the indented Ta_3_ZrB_8_ solid solution film by the focused ion beam (FIB) technique as illustrated in Fig. 3A1 and A2. The sample of the Ta_3_ZrB_8_ solid solution film was mounted on SEM stubs, and the selected indented area was coated with a layer of Pt metal to increase conductivity to enhance imaging quality and reduce damage to film surface by the Ga^+^ ion beam used for FIB cutting. To reduce the damage to the microstructure morphology of the cross-sectional sample, the Ga^+^ ion beam energy was selected to be 2 keV [25]. Ion beams etched grooves through the film thickness on both sides of the transverse axis of the indentation. Subsequently, along the direction of the white arrow in Fig. 3A2, the indentation cross-section was milled to a thickness of less than 30 nm to improve the transmittance during TEM characterization (Fig. 3A3). The TEM image of the cross-sectional sample is shown in Fig. 3B1, and a circular region with a diameter of about 126 nm beneath the indentation surface was selected for selected-area electron diffraction characterization (Fig. 3B1), which shows diffraction spots for the hexagonal crystal structure [26], in agreement with the structural analysis by the XRD measurements.

To further examine the impact of indentation loading on the crystal structure of the Ta_3_ZrB_8_ solid solution film, we took a close examination of an area directly underneath the indented surface. The HRTEM image of the cross-sectional sample (Fig. 3B2) reveals high-quality crystallinity without visible structural defects. Moreover, it is noted that the indented Ta_3_ZrB_8_ solid solution has a (001) interplanar spacing *d* of 0.326 nm, which is notably smaller than the corresponding (001) interplanar spacing *d* value of 0.343 nm taken at a site away from the indented surface (Fig. S2). This result shows that upon the elastic recovery after the removal of the indenter, the Ta_3_ZrB_8_ solid solution structure remains partially compressed but retains a high degree of crystallinity, which indicates that the strengthening and toughening observed in the experiments stem from intrinsic bonding characters of the chemically tuned solid solution structure.

To support the scenario of intrinsic strengthening and toughening in Ta_3_ZrB_8_ solid solution, we further examined factors that may influence the hardness and toughness of the film samples, including residual compressive stress in the film, grain size, tissue phase around grain boundaries, indentation-induced structural transformation, and dislocation generation and movement. Residual compressive stress in film samples tends to enhance measured hardness [27]. We calculated the intrinsic stress of as-deposited films using the Stoney equation [28] and obtained −2.99 ± 0.23, −1.02 ± 0.16, and −1.39 ± 0.11 GPa for the TaB_2_, Ta_3_ZrB_8_, and ZrB_2_ films, respectively. It is seen that the Ta_3_ZrB_8_ solid solution film hosts the lowest intrinsic compressive stress yet the highest measured hardness, suggesting that compression stress is not a determining factor for the strengthening of Ta_3_ZrB_8_ solid solution over TaB_2_ or ZrB_2_. Highly crystallized and oriented film specimens comprising grains with a size of 20 nm or larger are known to exhibit minimal grain-boundary effect and possess hardness close to that of high-quality single crystals [29]. The grain sizes for the synthesized TaB_2_, Ta_3_ZrB_8_, and ZrB_2_ films in this work are ~20, ~33, and ~28 nm, respectively. Therefore, grain size is not a main influencing factor in enhancing the mechanical properties of the Ta_3_ZrB_8_ solid solution film. It has been reported that a tissue phase may improve hardness; for example, a boron-rich tissue phase at the grain boundaries of TiB_2.4_ films has shown to contribute to notable improvement of hardness because the high cohesive strength of the tissue phase prevents grain-boundary sliding [30]. Moreover, such tissue phases may also help improve toughness via inhibition of crack propagation and promotion of grain-boundary sliding [31]. However, no tissue phase was found in the crystal structure of Ta_3_ZrB_8_ solid solution. Another possible source of structural toughening is phase transformation during indentation, which dissipates a large amount of deformation energy and deflects or even stops the crack propagation [32]. However, the results in Fig. 3B clearly show that there is no structural transformation in the Ta_3_ZrB_8_ solid solution film during the nanoindentation test, thereby removing this possibility. In addition, a recent work reported positive effect of stacking fault and twinning on the plastic deformation of interstitial intermetallic compounds [33]. In fact, for TMB_2_ with high crystalline quality, the material deformation mode at room temperature usually transitions from complete elasticity to suddenly fracture [7]; thus, there is probably no stacking fault and twinning-induced plasticity under the conditions reported in our present work. We finally evaluate the role of dislocation in affecting the mechanical properties. Dislocation pinning is a major mechanism for hardness improvement in metallic solid solutions, and dislocation slip dominates plasticity of metallic systems. However, for ceramics similar to TM compounds studied in this work, dislocation generation and motion are not major deformation modes due to the very high energy cost associated with the breaking and rearrangement of the strong (pure or mixed) covalent bonds. Instead, incipient plastic formation is usually dominated by brittle bond breaking, followed by crack propagation. We have analyzed the area underneath the indented surface using the selected-area inverse fast Fourier transform method, and the results in Fig. 3B3 show no dislocation in this area. Some literatures reported that TMB_2_ films with the (001) preferred orientation make it difficult to form dislocation [26,30], which hints that dislocation may not be a major relevant factor for solid solution strengthening and toughening in Ta_3_ZrB_8_ solid solution. Moreover, we likewise obtain the scanning TEM high-angle annular dark-field (STEM-HAADF) picture (Fig. 3C1) beneath the indentation surface, which illustrates the high crystalline quality of Ta_3_ZrB_8_. Subsequently, the corresponding geometric phase analysis is executed to observe the strain distribution of film after indentation test. The [0001] and [1010] are defined as *x* and *y* axes, respectively. The stain maps of horizontal normal strain (*e_xx_*) and vertical normal strain (*e_yy_*) are computed and displayed in Fig. 3C2 and C3, which exhibit the compression stain (blue color) along the direction perpendicular to (0001) plane and the tensile strain (red color) along the direction parallel to (0001) plane, indicating a complex strain state of Ta_3_ZrB_8_ under berkovich indentation deformation mode. Below, we carry out a systematic and in-depth theoretical evaluation of the structural deformation modes and stress responses of Ta_3_ZrB_8_ solid solution under indentation loading conditions, and the results are compared to those of TaB_2_ and ZrB_2_ to reveal the atomistic mechanisms responsible for the strengthening and toughening caused by the chemical tuning of bonding charge on the main load-bearing bonds in the solid solution crystal.

### Strengthening/toughening of Ta_3_ZrB_8_ solid solution: Theoretical evaluation

We checked the stability of the Ta_3_ZrB_8_ solid solution by evaluating its formation enthalpy and phonon spectrum. The obtained negative mixing enthalpy of −95.173 meV/atom indicates the energetic stability, and a lack of imaginary phonon modes in the entire Brillouin zone of Ta_3_ZrB_8_ (Fig. S3) shows the dynamic stability. These results help to explain the successful experimental synthesis of this compound and establish its robust viability for practical applications.

**Fig. 4.**
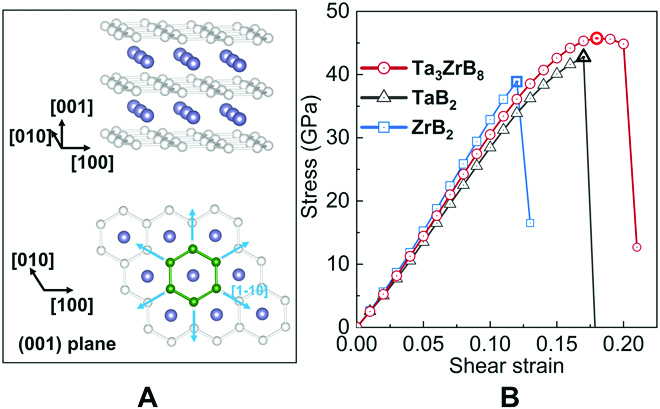
(A) Crystal structure of TMB_2_ with 6-fold symmetric bonding configuration in the (001) plane, as indicated by 6 equivalent [1-10] directions, where the small (large) spheres represent boron (TM) atoms. (B) Calculated stress–strain relations under the (001)[1-10] Berkovich indentation shear strain constrained by a normal stress for ZrB_2_, Ta_3_ZrB_8_, and TaB_2_.

We then performed stress–strain calculations for TaB_2_, ZrB_2_, and Ta_3_ZrB_8_ under indentation loading condition comprising a shear strain constrained by a coexisting loading-induced normal compression. Here, we focus on the case of indentation on the (001) oriented crystal, which is the preferred growth orientation of the synthesized film samples. The (001) crystal plane has a 6-fold bonding symmetry as illustrated in Fig. 4A for the [1-10] directions. We assessed that indentation strength by calculating normal compression constrained shear stress response to strains along the high-symmetry (001)[1-10] slip directions, where the evenly distributed dense bonding network serves to sustain the indentation deformation applied to specimens in the (001) orientation. The calculated peak stress of the corresponding stress–strain curve is defined as the indentation strength, which is directly related to measured hardness [34,35]. Our calculated results (Fig. 4B) show that the indentation strengths of the (001) oriented TaB_2_, ZrB_2_, and Ta_3_ZrB_8_ are 42.7, 38.8, and 45.7 GPa, respectively; moreover, the Ta_3_ZrB_8_ crystal exhibits extended strain range that is notably longer than those of TaB_2_ and ZrB_2_, signaling considerably improved ductility and toughness as measured by the area under the stress–strain curves [36]. These results show that intrinsic stress–strain relation of the Ta_3_ZrB_8_ solid solution can produce the long-sought simultaneous strengthening and toughening, which deviates considerably from Vegard’s law that describes properties of a solid solution via a linear interpolation relation between the respective properties of the constituent pure materials [37]. In addition, the indentation stress–strain relation in the (001)[110] slip direction was also evaluated (Fig. S4), where the peak stress for TaB_2_, ZrB_2_, and Ta_3_ZrB_8_ are 42.9, 35.6, and 40.3 GPa, respectively, which support the main conclusion of this work. Moreover, it is noted that the stronger resistance in the [1-10] slip direction determines the indentation response of the specimen in the (001) orientation with a 6-fold crystal symmetry spanning a network of strong directions capable of resisting indentation deformation applied in the (001) plane, which is similar to the situation reported for OsB_2_ [38]. It needs to be emphasized that the results obtained from indentation stress–strain curve are usually in good agreement with the experimental hardness measured by well-controlled nanoindentation test. This calculation method obviously differs from the results derived from uniaxial shear strain–stress curve due to the difference of crystal deformation mode. The latter makes the crystal slip along a direction in the fixed crystal plane without normal compressive stress, which is usually unsuitable for direct comparison with indentation hardness measurements. For example, the uniaxial shear strength of ZrB_2_ along the (0001)[10-10] slip direction, which is equivalent to the (001)[1-10] slip direction, is 46.3 GPa [39], while the biaxial indentation strength of ZrB_2_ along the (001)[1-10] slip direction in our work is 38.8 GPa, and this value is much closer to the indentation hardness of ~33.0 GPa.

**Fig. 5.**
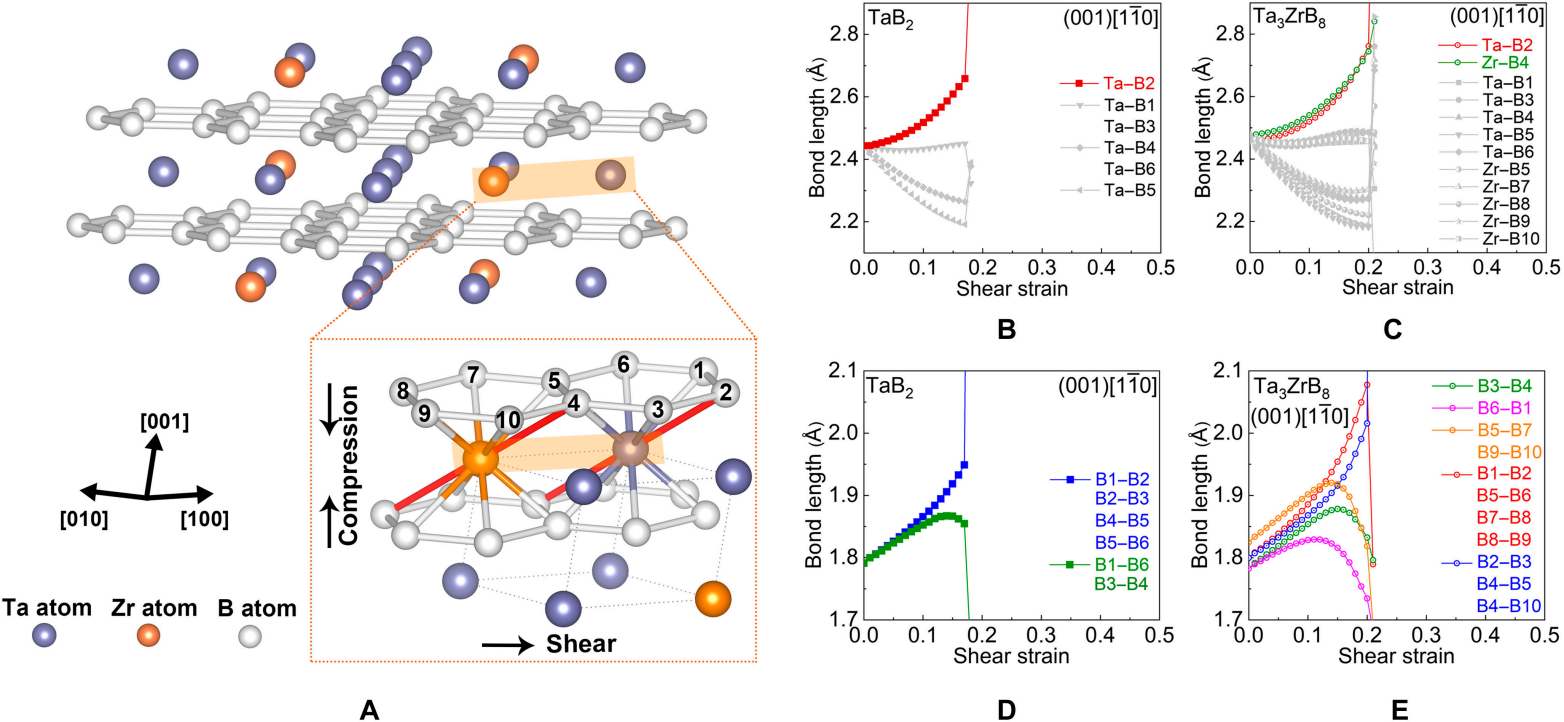
(A) Illustration of the change of bonding patterns in TaB_2_ and Ta_3_ZrB_8_ under the (001)[1-10] indentation; the compressive and shear strain directions are indicated by the black arrows. The thicker lines connecting atoms indicate the main load-bearing bonds that are lengthened under indentation. Also shown is the evolution of the lengths of (B and C) TM–B bonds and (D and E) B–B bonds in TaB_2_ and Ta_3_ZrB_8_. The main load-bearing bonds are highlighted by the colored symbols and lines, which include a small selected set of TM–B bonds and all the B–B bonds.

**Fig. 6.**
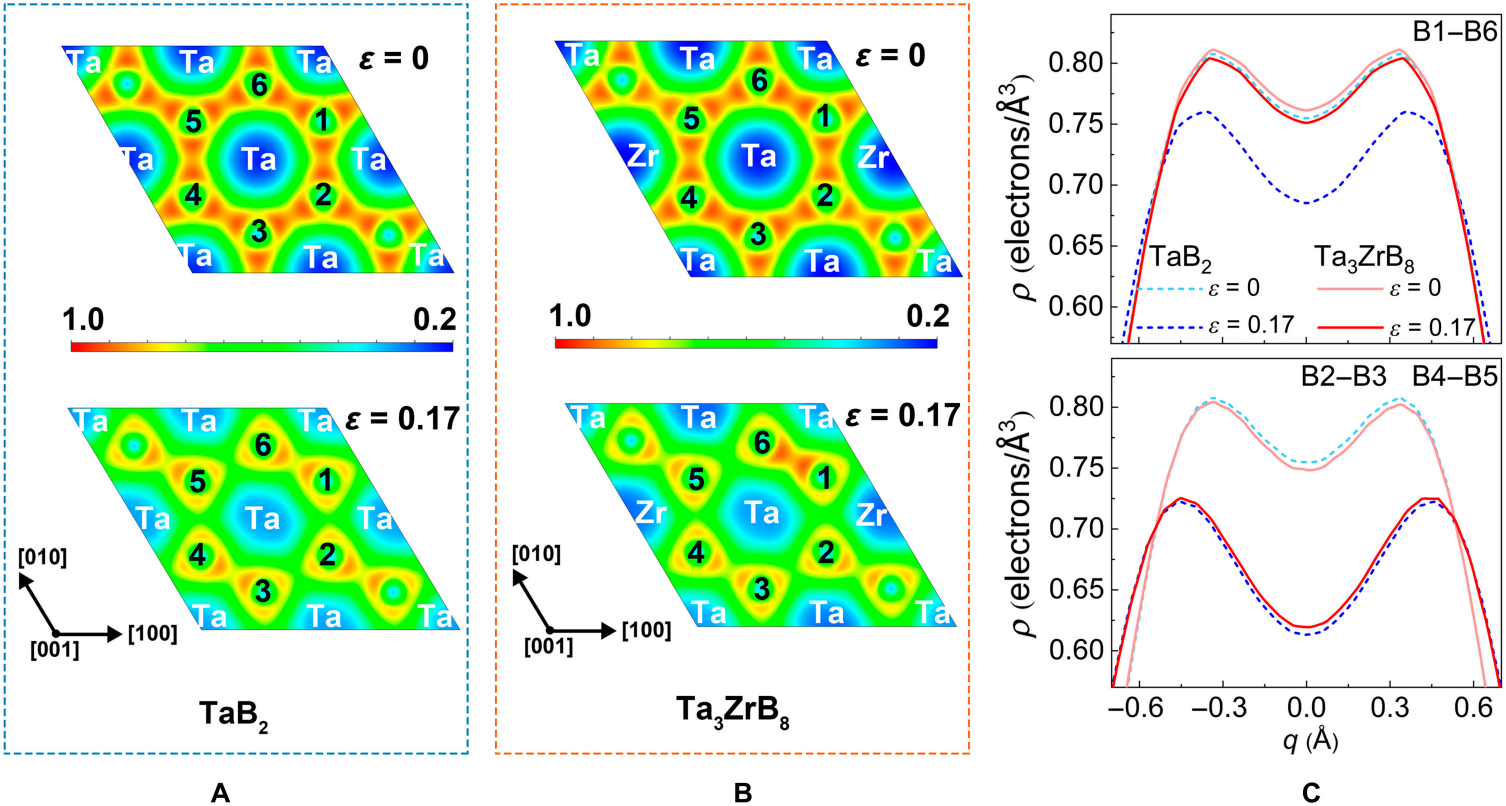
Electron density distribution of the boron atom layer in the (001) plane of (A) TaB_2_ and (B) Ta_3_ZrB_8_ under indentation shear strains of ε = 0 and 0.17 as indicated. The colored scale units are in electrons per cubic angstrom. (C) Comparison of the electron density distribution along different B–B bonds in TaB_2_ and Ta_3_ZrB_8_ under indentation shear strains at equilibrium (ε = 0) and at ε = 0.17.

We further examined the evolution of bond lengths and bonding charge under the indentation deformation to unveil the mechanism for the strengthening and toughening of Ta_3_ZrB_8_ solid solution. Analysis of the bond length variations in deformed Ta_3_ZrB_8_ (Fig. 5) shows that all the B–B bonds along with a small set of TM–B bonds are the main load bearers under the indentation shear strains, because these bonds exhibit the largest elongation in response to the strains and are therefore responsible for setting the limit on the load-bearing capacity of the material. Consequently, we focus on the charge evolution in the boron layer. Results (Fig. 6 and Fig. S5) show that at equilibrium, there is no obvious difference in the electron distribution along the B–B bonds between TaB_2_ and Ta_3_ZrB_8_; however, under rising strains, a deformation-induced charge redistribution develops along the B–B bonds. In solid solution Ta_3_ZrB_8_, the difference of the charge distributions along all B–B bonds under large strain becomes more pronounced. In every direction of the boron 6-membered ring, there are some enhanced B–B bonds in the solid solution, compared to the base compound TaB_2_. Selected B–B bonds in the [210] and [120] directions retain more electron under large strains in Ta_3_ZrB_8_, and some B–B bonds in the [1-10] direction are much less charge depleted under large strains after Zr doping, leading to a more than 20% strain range increase from 0.17 in TaB_2_ to 0.21 in Ta_3_ZrB_8_, generating the enhanced peak stress and related strain range. We also examined the charge distribution on the TM–B bonds due to their crucial role in sustaining the 3-dimensional TMB_2_ crystal structure. As shown in Fig. S6, the Ta–B load-bearing bonds along the slip direction in Ta_3_ZrB_8_ are enhanced compared to those in TaB_2_, contributing to the strengthening and toughening of the solid solution.

The bonding charge redistribution caused by introducing the solute atom (Zr) with an EN in proper contrast with that of the solvent atom (Ta) enhances the main load-bearing B–B bonding network while still retaining sufficiently strong TM–B bonding network, thereby extending the strain range and enhancing the peak stress, leading to the concurrent strengthening and toughening of Ta_3_ZrB_8_ solid solution. It is noted that there exist many other parameters that affect mechanical properties, and, among them, valence electron concentration (VEC) is the most influential factor to regulate the mechanical properties of materials [40]. Recently, VEC is also shown to be a convincing indicator to regulate the mechanical properties of TMB_2_ by checking the elastic constants (*C_ij_*), bulk modulus (*B*), and shear modulus (*G*). Specifically, theoretical hardness (*H_Tian_*) and brittleness are enhanced as VEC decreases from a broad perspective. However, within a narrow range of VEC, the elastic constants for different systems are often scattering widely with following a regular distribution pattern, which suggests the need to introduce a secondary yet significant factor, such as EN. In our present work, we examined the values of key parameters underlying the mechanical properties for the 3 samples to evaluate the effect of VEC variation, as shown in the Table. It is seen that Zr atoms introduced into TaB_2_ make the VEC go down by 0.25 compared with TaB_2_. Among these quantities, *C*_11_ and *C*_33_ measure a material’s ability to resist linear compressions along different crystallographic axes [41], and *B* represents the material’s ability to resist volume change [42]; these parameters of Ta_3_ZrB_8_ are between the values of ZrB_2_ and TaB_2_, indicating an interpolation relation of volume deformation resistance. Moreover, *C*_44_ and *G* are related to the material’s ability of shear resistance [19], and Ta_3_ZrB_8_ hosts weak shear resistance owing to its small *C*_44_ and *G*. The ductility for Ta_3_ZrB_8_ has a slight improvement inferred from its small *G*/*B* despite the intrinsic brittle feature of the samples. Hence, VEC has a visible impact on these key material parameters. Nevertheless, the theoretical hardness values obtained using Tian’s model based on elastic moduli exhibit the variation trend of ZrB_2_ > Ta_3_ZrB_8_ > TaB_2_, which is evidently different from the experimental hardness variation trend and is better described by considering the effect of EN and indentation strain–stress relations. This situation highlights 2 important points: (a) EN plays a more significant role in regulating the mechanical properties of Ta_3_ZrB_8_ solid solution compared to the variation of VEC within a narrow range caused by the Ta/Zr substitution; (b) compared with commonly used *C_ij_*, indentation stress–strain relations provide a more appropriate foundation for evaluating the mechanical responses probed by nanoindentation. These findings expand the traditional solid solution approach to a new realm of chemically tuning strong covalent bonding materials. More importantly, this strengthening and toughening solid solution protocol constructed by introducing metal atoms with relatively low EN is also effective in other material systems. For instance, the EN of Zr (1.33) is lower than that of Ti (1.54), and as Zr atoms are doped into TiN lattice, the Ti_1−*x*_Zr*_x_*N (*x* = 0.41) film exhibits a slightly higher hardness and *H*^3^/*E*^2^ value than the corresponding results for TiN [43]. Similar phenomena have been reported in MoVN [44] and CrVN [45], which show a strengthening and toughening effect, as V atoms with relatively lower EN are introduced into parent TMN (Mo_2_N or CrN) phases. The crystal structure and chemical bonding of TMN (or TMC) are different from those of TMB_2_ in that TMN (or TMC) usually adopts the rock-salt structure with TM–TM metallic bonds and TM–N (or TM-C) ionic bonds, while, in contrast, TMB_2_ crystallizes in hexagonal structure with alternately stacked metal layers and B layers, consisting of not only the TM–TM metallic bonds and TM–N (or TM–C) ionic bonds but also B–B covalent bonds. Considering that the B–B covalent bonds are the strongest among all chemical bonds in TMB_2_, when the EN of TM is changed, the reinforced B–B covalent bonds produce more marked positive effects on the strength and toughness of TMB_2_, although the optimized TM–B ionic bonds also contribute. For TMN, the change of EN only regulates the TM–N ionic bonds, which are similar to the TM–B bonds in this work, and the solute metal atom with lower EN in TMN may also improve the TM–N ionic bonds between solvent metal and N atoms, leading to the strengthening and toughening phenomena in TMN systems. Furthermore, we also notice that the EN of B (2.04) is much lower than that of N (3.04), and as a result, the EN deviation between metal and B atoms is smaller than that between metal and N atoms. Hence, B atoms are more sensitive to the variation of the EN of metal atoms than N atoms; as a result, the optimization of TM–N bonds may be inferior compared to TM–B bonds. Even so, the EN of solute metal atoms may still play an effective role in regulating broader classes of materials.

**Table.** The VEC, elastic constants *C*_11_, *C*_33_, and *C*_44_ (in gigapascals), bulk modulus *B* (in gigapascals), shear modulus *G* (in gigapascals), Pugh’s ratio *G*/*B*, Tian’s hardness *H_Tian_* (in gigapascals), maximum strain *ε* of indentation strain–stress curve, and peak stress *σ* of indentation strain–stress curve for the 3 samples studied in this work.

	VEC	*C*_11_	*C*_33_	*C*_44_	*B*	*G*	*G*/*B*	*H_Tian_*	*ε*	*σ*
TaB_2_	11.00	629.3	489.1	235.6	308.5	231.9	0.78	31.5	0.17	41.6
Ta_3_ZrB_8_	10.75	614.3	476.3	219.1	295.6	216.7	0.73	29.1	0.21	45.7
ZrB_2_	10.00	571.7	452.3	245.2	246.9	235.2	0.95	41.5	0.12	38.8

## Conclusion

We have carried out a joint experimental and computational study to establish a new realm for solving classic strength–toughness tradeoff dilemma for materials comprising strong covalent bonds, using TMB_2_ TaB_2_ as a material template for a case study. We have developed a chemically tuned solid solution approach by constructing the dual-TMB_2_ Ta_3_ZrB_8_ via the introduction of Zr to partially substitute for Ta in the original TaB_2_ compound. The lower EN of Zr atoms relative to that of Ta atoms weakens the ability of Zr atoms to attract charge during indentation-induced shear deformation, creating a new charge map that rebalances the bonding patterns among the main load-bearing B–B and TM–B bonds for improved overall stress and strain responses under the indentation loads. These considerations are realized in systematic experimental synthesis and characterization and elucidated via in-depth computational simulations and analysis. The measured hardness of the synthesized films of TaB_2_ (45.9 ± 1.0 GPa) and ZrB_2_ (33.0 ± 1.7 GPa) is notably enhanced in the solid solution Ta_3_ZrB_8_ (49.5 ± 2.2 GPa), which also possesses enhanced toughness as indicated by the absence of indentation-induced cracks. These findings validate our design rationale for solving the strength–toughness dilemma in superhard TMB_2_ via a distinct chemically tuned solid solution approach, thereby opening a new avenue for exploring optimal hardness–toughness balance in wide-ranging strong covalent materials.

## Materials and Methods

### Film deposition and characterization

The TaB_2_ (ZrB_2_) film was grown on a double-side polished Al_2_O_3_ (0001) substrate using a TaB_4_ (ZrB_4_) target (60 mm in diameter, 99.95% pure) in a direct current (DC) magnetron sputtering system with a base pressure of 5 × 10^−4^ Pa. Growth took place in Ar (99.999% pure) gas environment at a constant DC power of 200 W for 180 min; meanwhile, the target-to-substrate separation, work pressure, substrate temperature, substrate bias voltage, and substrate rotation speed were set at 8 cm, 0.8 Pa, 600 °C, −80 V and 10 rpm, respectively. The Ta_1−*x*_Zr*_x_*B_2_ solid solution films were deposited by magnetron cosputtering individual TaB_4_ and ZrB_4_ targets. The DC power for the ZrB_4_ target remained 200 W, while the DC power for the TaB_4_ target was set at 150 W, and the other sputtering parameters were kept the same as those described above. The film thicknesses are ~1.1, ~1.6, and ~2.6 μm, respectively. Chemical contents for as-deposited films were characterized by x-ray photoelectron spectroscopy (ESCALAB-250) using Al Kα as the x-ray source. The x-ray photoelectron spectroscopy results show that the ratio of Ta and Zr content in the synthesized Ta_1−*x*_Zr*_x_*B_2_ film is 3:1. The *θ*-2*θ* XRD scans used a Bragg–Brentano (Bruker D8) diffractometer with a monochromatic Cu Kα source (*λ* = 0.15418 nm) to characterize the crystal structure of the as-deposited films. The measurement was performed in locked coupled mode with 40-mA current and 40-kV voltage. Readings were recorded at a scan speed of 0.5 s/step in a 2*θ* range from 20° to 60°. For the obtained solid solution film, the indentation cross-section was fabricated using an FIB system (FEI Strata 400S) with a coarse milling condition of 30 keV and a final polishing condition of 2 keV. The HRTEM (JEOL 2010F and JEM 2100F), operated at 200-kV accelerating voltage, was utilized to confirm the crystal structure of the samples. The aberration-corrected STEM (Titan Cubed Themis G2300) was used to obtain the HAADF picture of atomic phase of films. Nanoindentation tests were performed by an MTS Nano Indenter G200 system equipped with continuous stiffness measurement mode [46]. During nanoindentation test, the loading function was displacement controlled with a constant rate of 10 nm/s. A maximum indent depth of 600 nm for each film was reached by a Berkovich pyramidal diamond probe (apex angle = 142.3°) with a nominal tip radius of 150 nm, and the fused quartz standard of known hardness and elastic modulus was used as calibration [23]. Nine load–displacement curves were acquired in each film and analyzed following the Oliver and Pharr method to obtain the hardness values. The distance of each indentation was set at 30 μm to avoid the effect of plastic deformation around the indentation [47]. The morphology of indentation of film was observed by an SEM (Hitachi SU8010) at 2-kV high voltage.

### Density functional theory calculations

Structural optimization of the TMB_2_ compounds in *P*6*/mmm* symmetry (no. 191) was performed using Vienna Ab Initio Simulation Package code [48], using the periodic boundary conditions, projector-augmented wave potentials [49], and the Perdew–Burke–Ernzerhof (PBE) generalized gradient approximation [50] for exchange correlation energy with a plane-wave basis set. For TaB_2_, ZrB_2_, and Ta_3_ZrB_8_ of 2 × 2 × 2 supercell, the conjugate gradient optimization method was employed to relax atomic positions and structural parameters, with the total energy of the structure converged to 10^−6^ eV/atom and residual forces on each atom less than 0.005 eV/Å. A 20 × 20 × 20 Monkhorst–Pack *k*-point grid and a 500 eV cutoff energy were used in the calculations to determine the structural optimization, charge distribution, and stress responses. Indentation strength was obtained from the calculated Berkovich stress–strain relationships. The stress–strain curves were obtained by fixing the applied strain along specific loading paths and relaxing the lattice vectors and atomic positions step by step, and the peak stress before structural failure gives the indentation strength [51,52]. During the stress–strain calculations, the shear (*σ_zx_*) and normal compressive (*σ_zz_*) stress component obey the relation *σ_zz_* = *σ_zx_*tan*ϕ*, where *ϕ* is the centerline-to-face angle of the indenter [53]. The stress–strain calculations have proved to be suitable for estimating indentation strengths of diverse TM light-element compounds, providing accurate description of strength and atomistic deformation modes [14,54–56]. We have adopted this approach in this work to assess the chemically tuned solid solution strengthening and toughening phenomena and mechanisms for dual-TMB_2_. The lattice dynamical property is evaluated by employing density functional perturbation theory with 96 atoms per cell. Elastic constants *C_ij_* were extracted by introducing small finite distortions on the lattice to determine the elastic tensor to extract elastic constants. The polycrystalline elastic modulus and polycrystalline shear modulus were calculated using the Voigt–Reuss–Hill approximation.

## Data Availability

The data used to support the findings of this study are available from the corresponding author upon request.

## Supplementary Materials

Supplementary Materials Fig. S1. The hardness curve of as-deposited films and fused quartz as a function of indentation depth. Fig. S2. The HRTEM picture of Ta_3_ZrB_8_ cross-section, which is far away from the indentation. Fig. S3. Calculated phonon dispersion curve for Ta_3_ZrB_8_. Fig. S4. The indentation strain–stress curves for ZrB_2_, TaB_2_, and Ta_3_ZrB_8_ samples in the (001)[110] slip direction. Fig. S5. The electron density distribution in the (110) plane of TaB_2_ and Ta_3_ZrB_8_ under *ε* = 0 and 0.17, as indicated. The colored scale units are in electrons per cubic angstrom. Fig. S6. (A) Illustration of the change of the bonding patterns in TaB_2_ and Ta_3_ZrB_8_ under the (001)[1-10] indentation; the compressive and shear strain directions are indicated by the black arrows. The thicker lines connecting atoms indicate the main load-bearing bonds that are lengthened under indentation. Also shown is (B and C) the comparison of the electron density distribution along different TM–B bonds in TaB_2_ and Ta_3_ZrB_8_ under indentation shear strains at equilibrium (*ε* = 0) and at *ε* = 0.17.
