# Supplementary material for: Solving Strength–Toughness Dilemma in Superhard Transition-Metal Diborides via a Distinct Chemically Tuned Solid Solution Approach
Source: Research (Wash D C). 2023 Jan 16;6:0035. doi: 10.34133/research.0035 (PMC10076038; doi:10.34133/research.0035)
Supplement: Supplementary Materials — Fig. S1. The hardness curve of as-deposited films and fused quartz as a function of indentation depth. Fig. S2. The HRTEM picture of Ta3ZrB8 cross-section, which is far away from the indentation. Fig. S3. Calculated phonon dispersion curve for Ta3ZrB8. Fig. S4. The indentation strain–stress curves for ZrB2, TaB2, and Ta3ZrB8 samples in the (001)[110] slip direction. Fig. S5. The electron density distribution in the (110) plane of TaB2 and Ta3ZrB8 under ε = 0 and 0.17, as indicated. The colored scale units are in electrons per cubic angstrom. Fig. S6. (A) Illustration of the change of the bonding patterns in TaB2 and Ta3ZrB8 under the (001)[1-10] indentation; the compressive and shear strain directions are indicated by the black arrows. The thicker lines connecting atoms indicate the main load-bearing bonds that are lengthened under indentation. Also shown is (B and C) the comparison of the electron density distribution along different TM–B bonds in TaB2 and Ta3ZrB8 under indentation shear strains at equilibrium (ε = 0) and at ε = 0.17. [file research.0035.f1.docx]

**Supplementary Information**

**for**

**Solving Strength-Toughness Dilemma in Superhard Transitional-Metal Diborides via a Distinct Chemically Tuned Solid Solution Approach**

Xinlei Gu^1^, Chang Liu^2^, Xinxin Gao^1^, Kan Zhang^1^, Weitao Zheng^1^, Changfeng Chen^3^

^1^State Key Laboratory of Superhard Materials, Department of Materials Science and Key Laboratory of Automobile Materials, MOE, Jilin University, Changchun 130012, China.

^2^International Center for Computational Methods & Software, College of Physics, Jilin University, Changchun 130012, China.

^3^Department of Physics and Astronomy, University of Nevada, Las Vegas, Nevada 89154, USA.

Correspondence should be addressed to Kan Zhang; kanzhang@jlu.edu.cn

This supporting file includes:

- Supporting Figure S1-S6


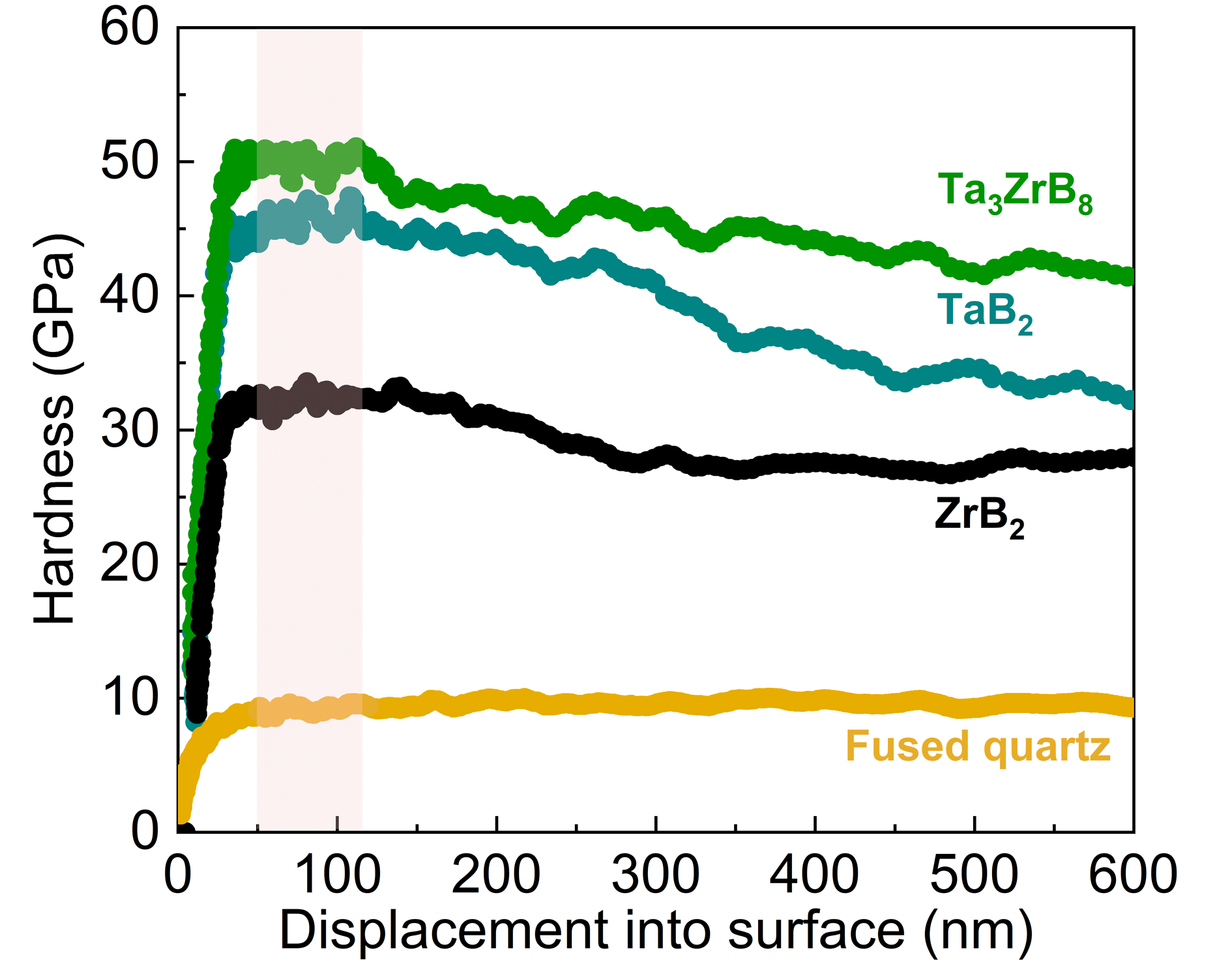


**Figure S1.** The measured hardness-displacement curves of the three as-deposited films, which are compared with the results on fused quartz, as a function of indentation depth.

The hard-displacement curves obtained from nanoindentation test in CSM mode can be utilized for estimating the hardness of films. To ensure accuracy of nanoindentation tests, fused silica is used as a calibration with its hardness remaining at ~10 GPa after the initial rise. The measured results for the TaB_2_, Ta_3_ZrB_8_, and ZrB_2_ films are shown here. To avoid the effect of surface ^1^ and substrate effect ^2^ during the nanoindentation test, the hardness readings from selected depths are usually used, in the 50-120 nm range in the present cases. Subsequently, the hardness values of TaB_2_, Ta_3_ZrB_8_, and ZrB_2_ films are obtained as 45.9±1.0, 49.5±2.2, and 33.0±1.7 GPa, respectively, which are consistent with data from the load-displacement curves shown in Figure 2(a).


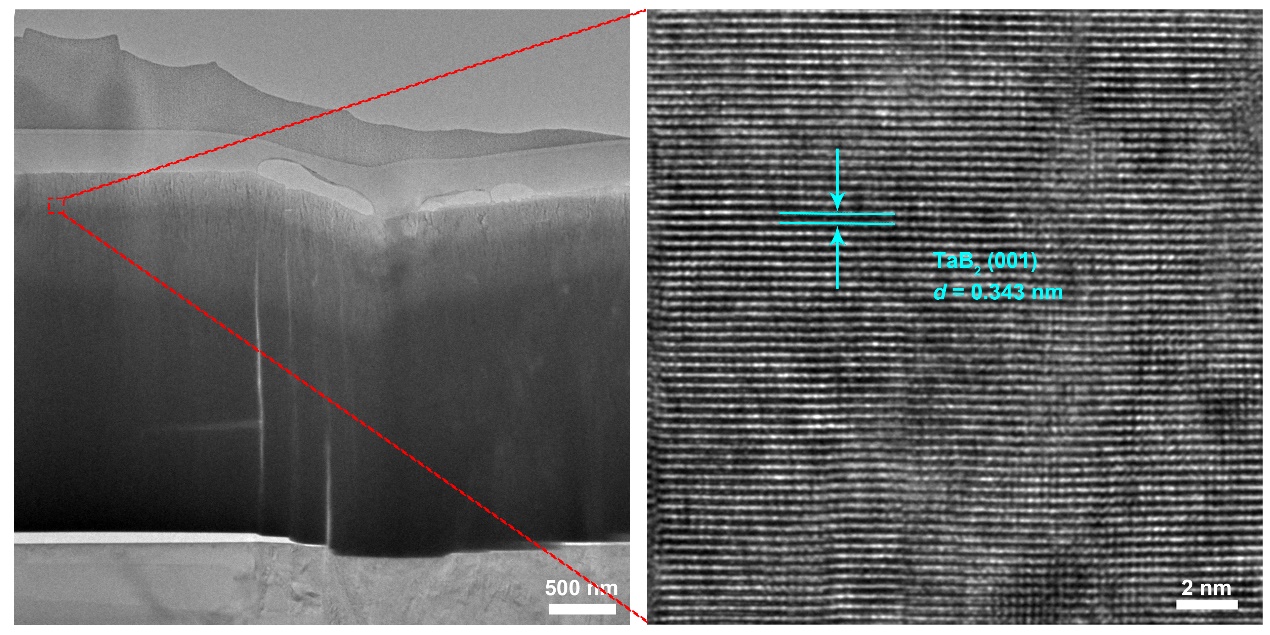


**Figure S2.** The HRTEM image of the Ta_3_ZrB_8_ film cross section taken from a region that is far away from the indentation site.


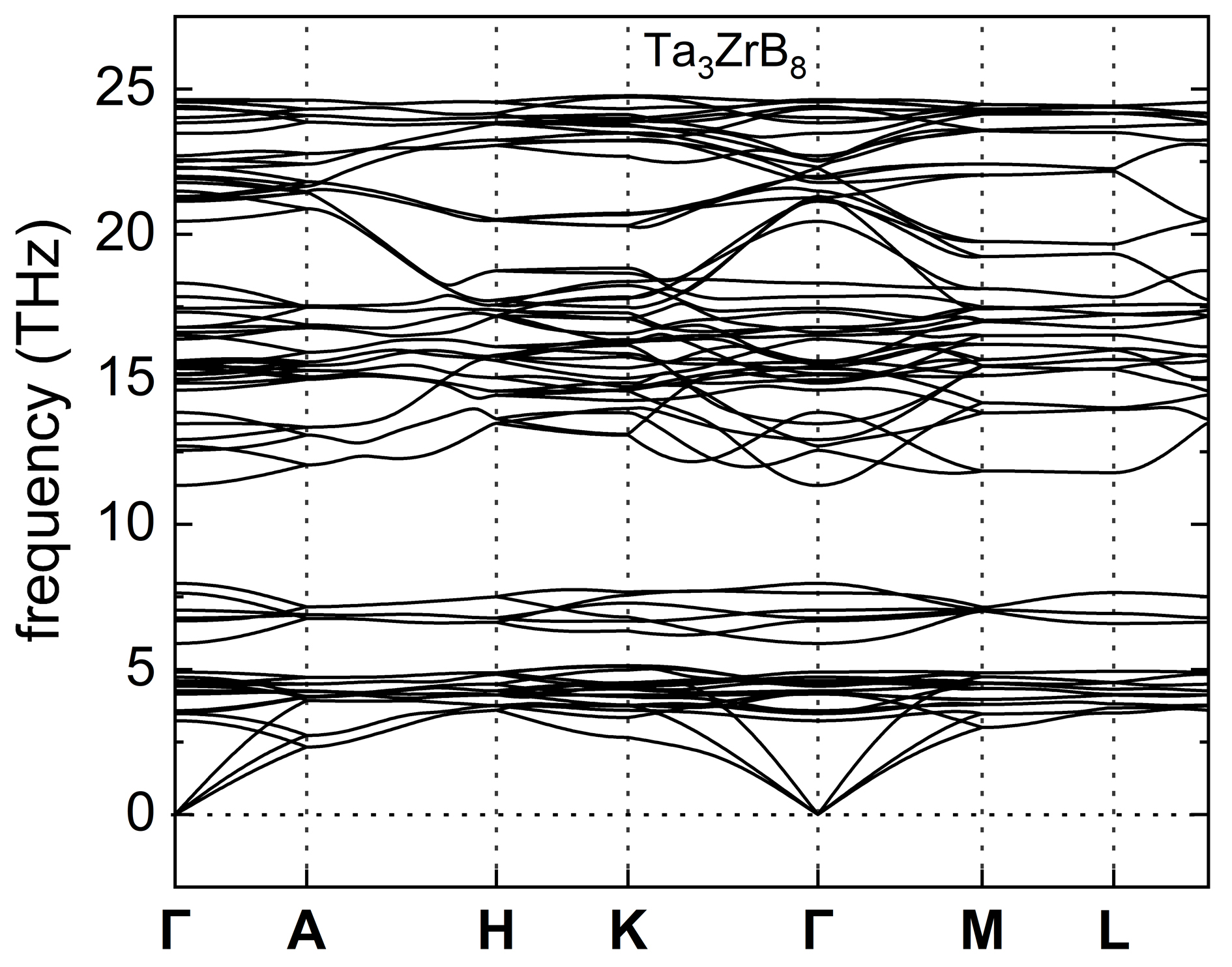


**Figure S3.** Calculated phonon dispersion curve for Ta_3_ZrB_8_.


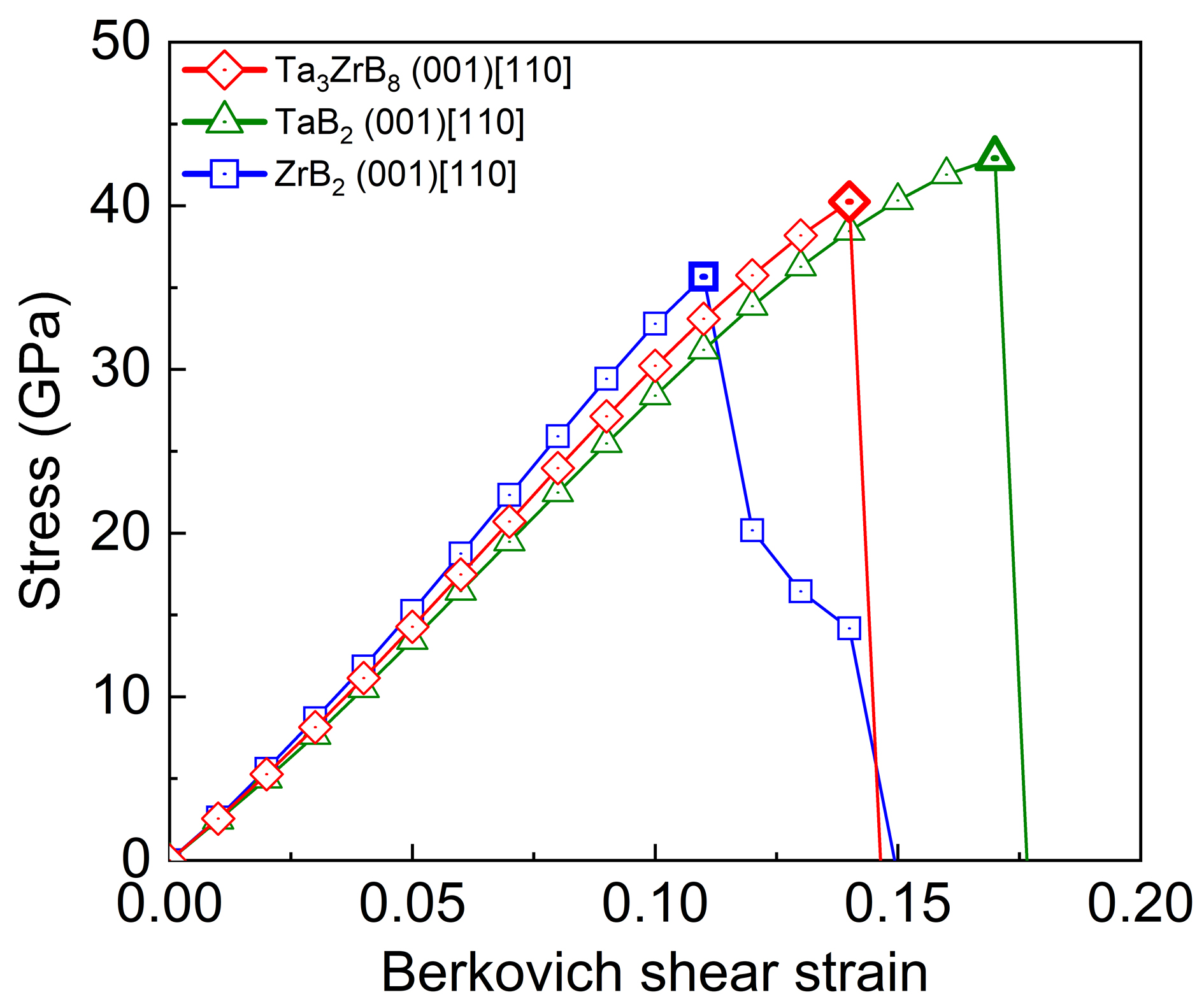


**Figure S4.** The indentation strain-stress curves for ZrB_2_, TaB_2_ and Ta_3_ZrB_8_ samples in the (001)[110] slip direction.


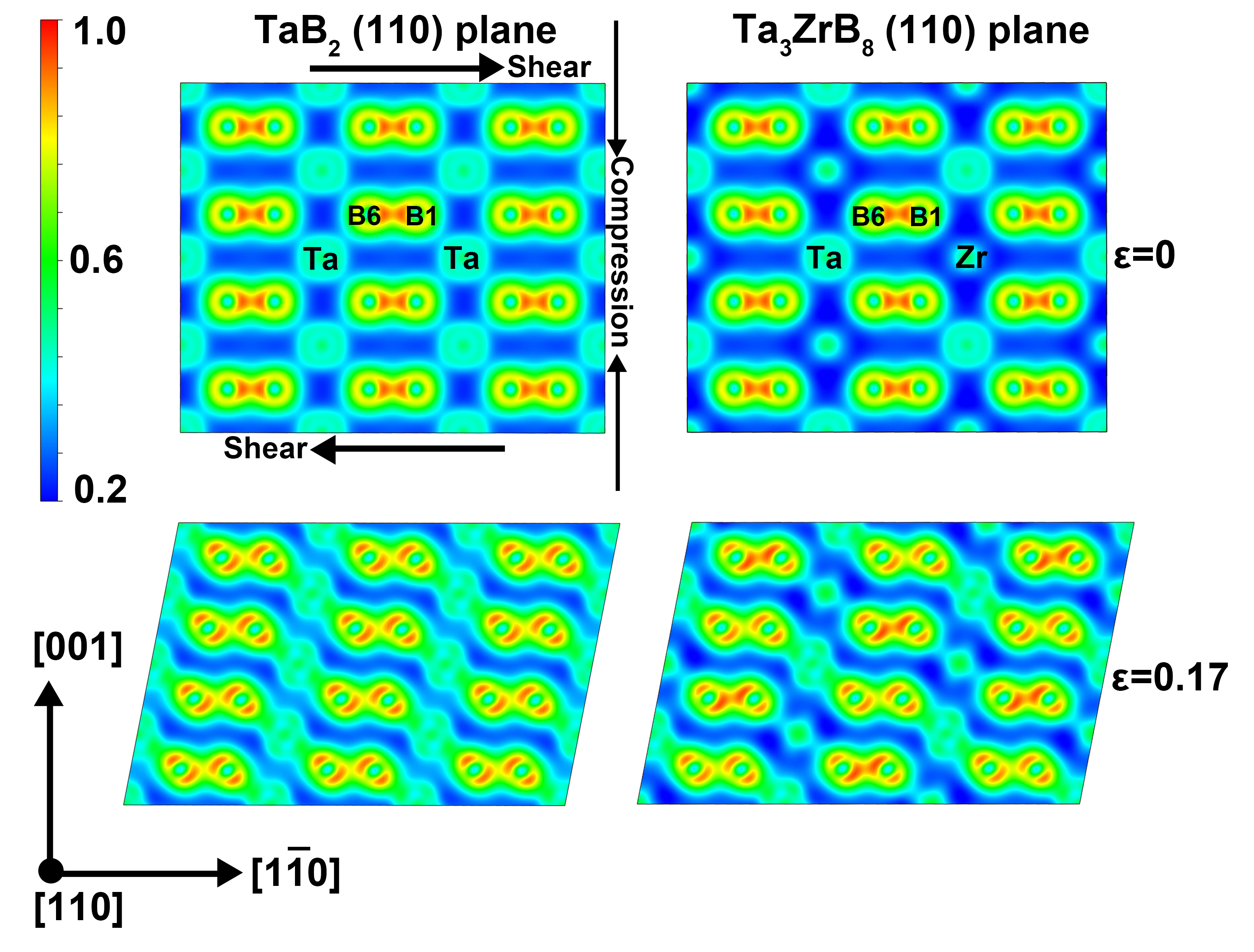


**Figure S5.** The electron density distribution in the (110) plane of TaB_2_ and Ta_3_ZrB_8_ under ε = 0 and 0.17, as indicated. The colored scale units are in electrons/Å^3^.


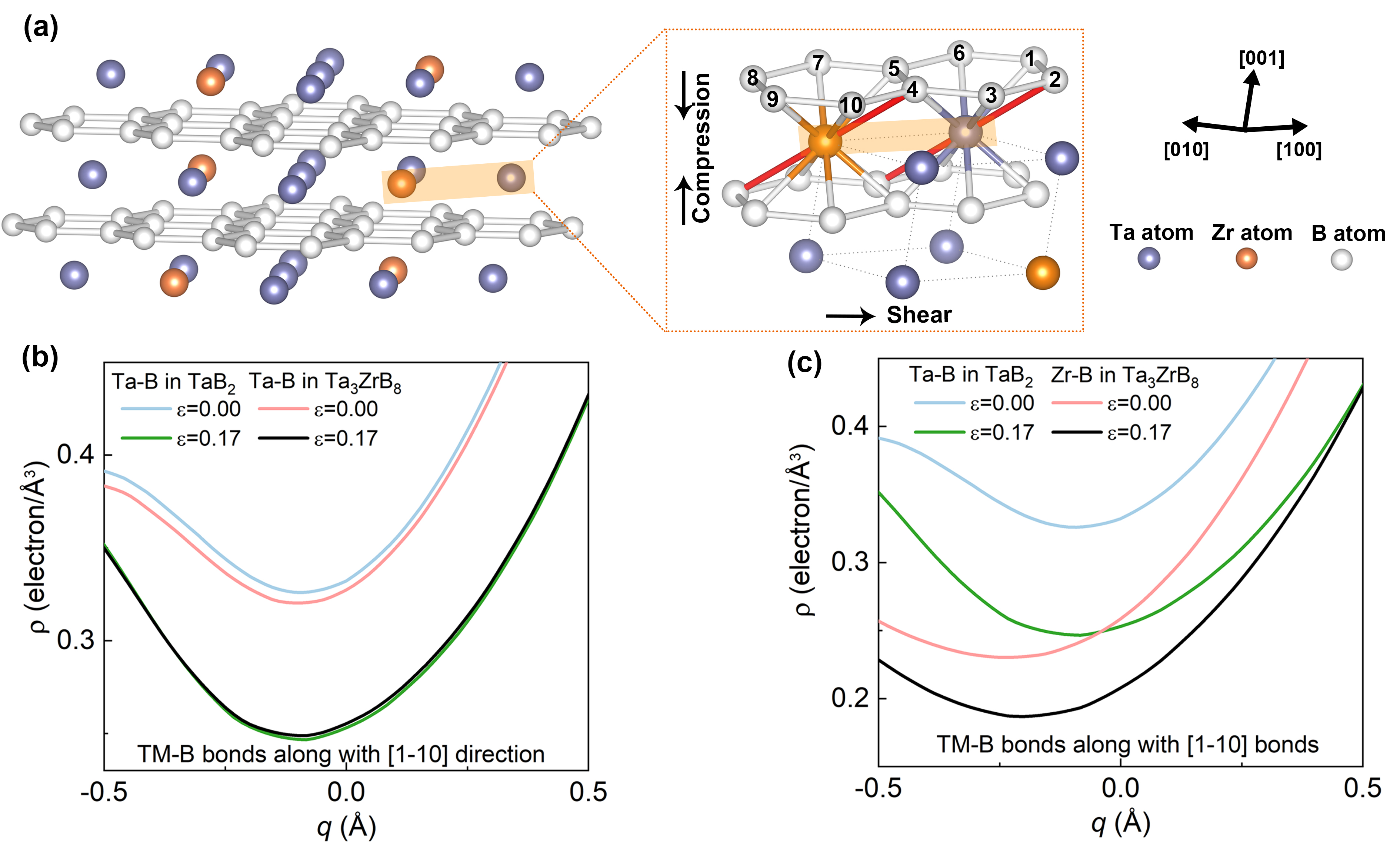


**Figure S6.** (a) Illustration of the change of the bonding patterns in TaB_2_ and Ta_3_ZrB_8_ under the (001)[1-10] indentation; the compressive and shear strain directions are indicated by the black arrows. The thicker lines connecting atoms indicate the main load-bearing bonds that are lengthened under indentation. Also shown are (b,c) the comparison of the electron density distribution along different TM-B bonds in TaB_2_ and Ta_3_ZrB_8_ under indentation shear strains at equilibrium (ε=0) and at ε=0.17.

Reference

1. Zhang, T. Y., and Xu, W. H. (2002) Surface effects on nanoindentation, J. Mater. Res. *17,* 1715-1720.

2. Saha, R. and Nix, W. D. (2002) Effects of the substrate on the determination of thin film mechanical properties by nanoindentation, Acta Mater. *50,* 23-38.
